# Glycemic impact of cereal and legume-based bakery products: Implications for chronic disease management

**DOI:** 10.1016/j.fochx.2024.101959

**Published:** 2024-11-01

**Authors:** Hiba Naveed, Waleed Sultan, Kanza Aziz Awan, Aysha Imtiaz, Sanabil Yaqoob, Fahad Al-Asmari, Ahmad Faraz, Jian-Ya Qian, Aanchal Sharma, Robert Mugabi, Saqer S. Alotaibi, Gulzar Ahmad Nayik

**Affiliations:** a Department of Food Science and Technology, Faculty of Science and Technology, University of Central Punjab, Pakistan; b School of Food Science and Engineering, Yangzhou University, Yangzhou, Jiangsu 225127, China; c School of Food and Biological Engineering, Jiangsu University, Zhenjiang, China; d Department of Food and Nutrition Sciences, College of Agriculture and Food Sciences, King Faisal University, Saudi Arabia; e University Centre for Research and Development, Chandigarh University, Gharuan, Mohali 140413, Punjab, India; f Department of Food Technology and Nutrition, Makerere University, Kampala, Uganda; g Department of Biotechnology, College of Science, Taif University, P.O. Box 110099, Taif 21944, Saudi Arabia; h Marwadi University Research Centre, Department of Microbiology, Marwadi University, Rajkot, Gujarat 360003, India

**Keywords:** Cereals, Legumes, Functional foods, Composite flours, Glycemic index, Disease management, Bakery products

## Abstract

This review examines the glycemic impact of cereal and legume-based bakery products and their potential role in chronic disease management, particularly in type II diabetes and cardiovascular diseases. The primary objective is to assess the glycemic index (GI) and glycemic load (GL) of bakery products made from cereals such as wheat and barley, and legumes like chickpeas, and to explore their effects on postprandial blood glucose response. Cereal-based products typically exhibit higher GIs (55–80), while legume-based bakery products demonstrate lower GIs (40–50), potentially contributing to better glycemic control. Incorporating legumes into bakery formulations can lower their glycemic index by up to 25 %. Legume-enriched bakery products may effectively manage blood glucose and reduce chronic disease risks like diabetes. However, more long-term studies are needed to confirm their broader benefits. This review emphasizes the need for innovation to improve the nutritional and sensory appeal of functional foods.

## Highlights

• Cereal and legume-based flours lower the glycemic index of bakery products by 20–30 %.
• These flours enhance insulin sensitivity, lower cholesterol, and reduce inflammation, helping prevent chronic diseases
• Increased fiber promotes satiety, supporting weight management and preventing obesity.
• Optimizing taste, texture, and consumer acceptance of these products remains a key area for future research.

## 1 Introduction

**Fig. 1:**
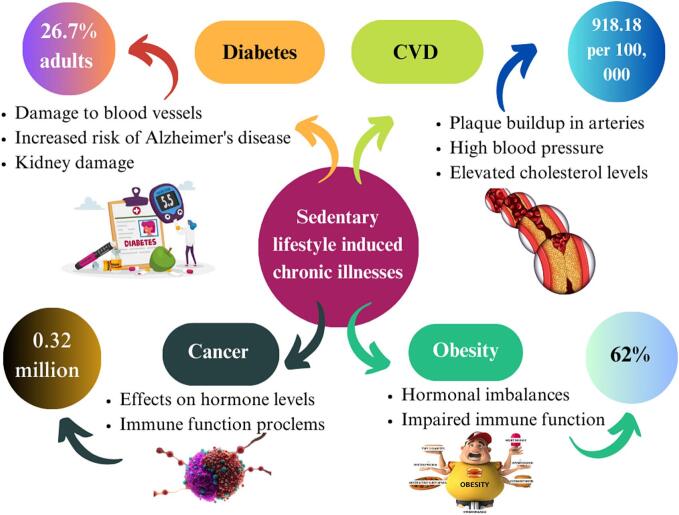
Prevalence of chronic diseases due to lifestyle.

Significant increases in the prevalence of physiological dysfunctions have been caused by sedentary lifestyles that are largely focused on imbalanced food consumption and insufficient physical activity, which are further exacerbated by the presence of environmental toxins (d'Angelo et al., 2019). In Pakistan, the rising consumption of hypercaloric foods, coupled with sedentary lifestyles, has led to a marked increase in diet-related disorders such as obesity, type II diabetes and cardiovascular diseases. These conditions, as illustrated in Fig. 1, are major contributors to severe health complications, including hypertension, metabolic syndrome, insulin resistance, and cardiovascular dysfunction, which collectively elevate morbidity and mortality rates across the population. According to data from the World Health Organization (WHO), approximately 200 million men and 300 million women globally are classified as obese, while an estimated 1.5 billion individuals are categorized as overweight (WHO, 2024). Overweight and obesity are contributing factors to the development of metabolic syndrome, that elevates the risk of diabetes, atherosclerosis, liver disease, brain disorders and cancer. All of these disorders exhibit a prevalent feature: a condition of mild inflammation caused by over-nutrition, that may be diagnosed at the molecular level (Hatch-McChesney & Smith, 2023).

Western diets are typically high in foods with a high glycemic index, such as cookies, pastries, and chocolate, which are strongly linked to the development of obesity and diabetes. These dietary patterns are also associated with chronic conditions, including diabetes, cancer, Alzheimer's disease, and cardiovascular disease, due to their role in elevating inflammatory biomarkers that contribute to the progression of these illnesses (Khemka et al., 2023). Saturated fatty acids (SFA) and industrially produced trans fats are commonly consumed in Western diets. Additionally, the low intake of long-chain polyunsaturated fatty acids (PUFAs) from the omega-3 series, such as EPA, DHA and ALA typically found in fish and plant sources further contributes to the high ω6/ω3 fatty acid ratio prevalent in Western diets. This imbalance is associated with various health risks, including inflammation and chronic diseases (Crawford et al., 2023). Non-food-related risk factors such as smoking, lack of physical activity and exposure to environmental pollutants that disrupt the endocrine system, also contribute to health issues. These factors, combined with unhealthy dietary and lifestyle choices, promote a chronic inflammatory state, which is a key indicator of the development and progression of chronic illnesses (Marruganti et al., 2023).

Extensive scientific evidence underscores the critical role of regular physical activity and a diet rich in vegetables, fruits and whole grains in the prevention and management of chronic diseases, particularly in developed nations (Wallace et al., 2020). Incorporating just one hour of daily exercise, combined with a nutrient-dense, fiber-rich diet, can significantly reduce the risk of conditions such as type II diabetes, metabolic syndrome, hypertension and certain cancers. Lifestyle approaches, such as the Pritikin program, have demonstrated great potential in preventing and even reversing chronic diseases, with benefits including improved lipid metabolism, better blood pressure regulation, enhanced insulin sensitivity, and reduced inflammation. These findings highlight the importance of further research to refine these intervention strategies and underscore the need to implement dietary and lifestyle changes as core approaches for chronic disease management (Cloete, 2023).

**Fig. 2:**
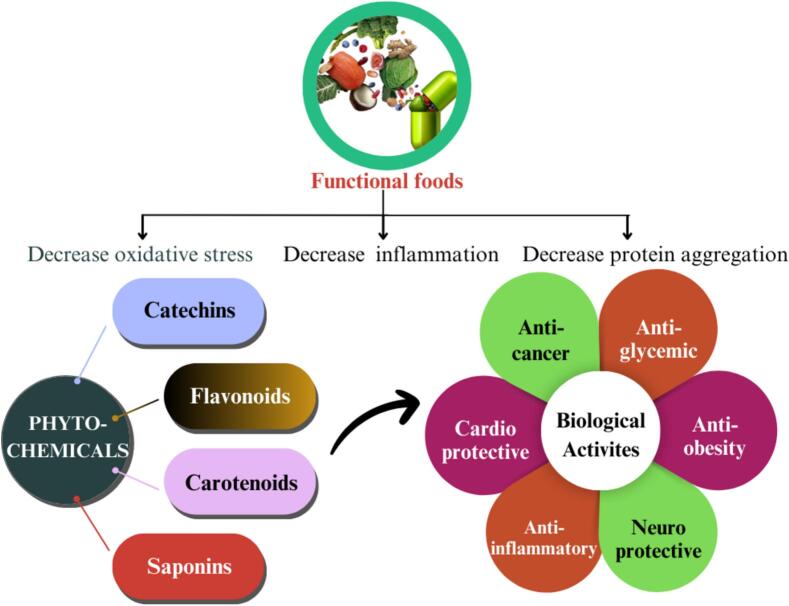
Benefits of functional foods: Phytochemicals and biological activities in chronic disease prevention.

Food encompasses the substances necessary for life-sustaining processes, including energy production, nutrient delivery, metabolic support and body maintenance (Izquierdo et al., 2021). In recent decades, there has been a growing interest in developing functional foods that offer health benefits beyond basic nutrition. The term “functional food” was introduced by Japan in the 1980s to describe products that not only provide nutrition but also contain ingredients aimed at supporting specific biological functions (Garud et al., 2023). Functional foods, such as those rich in antioxidants and with a low glycemic index (GI), help reduce postprandial oxidative stress, which is associated with various chronic disorders. The inclusion of phytochemicals and nutraceuticals in these products enhances individual health and reduces the risk of chronic diseases (Fig. 2) (Alongi & Anese, 2021). The global market for functional foods is rapidly expanding, currently exceeding $180 billion and growing at an annual rate of 8 % (Ali & Rahut, 2019). As demand increases, real-time monitoring is critical to ensuring the quality and safety of these products (W. Meng et al., 2023). To meet this demand, future innovation must balance scientific research with consumer expectations. A combined approach understanding market needs (market pull) and driving scientific advancements (science push) will offer the best chance for meaningful progress (Plattfaut & Koch, 2021). Scientific breakthroughs from fields such as genomics, proteomics, and sensor technology, along with insights from the Human Genome Project, have the potential to revolutionize health and nutrition (G. C. Wong et al., 2021). However, consumer acceptance remains essential, particularly as new biotechnologies enhance the functional food sector.

The glycemic index (GI) is a scale that measures how quickly carbohydrate-containing foods raise blood glucose levels after consumption, with rankings from 0 to 100. High-GI foods cause rapid spikes in blood sugar, while low-GI foods lead to slower, more gradual increases. The glycemic load (GL) provides a more accurate measure by considering both the GI and the carbohydrate content in a typical serving (Avedzi, 2019). While both GI and GL are crucial for managing blood sugar levels, recent evidence suggests that glycemic variability fluctuations in markers such as hemoglobin A1c (HbA1c) and fasting plasma glucose (FPG) plays a critical role in cardiovascular outcomes for patients with type 2 diabetes mellitus (T2DM). Notably, higher HbA1c variability has been linked to an increased risk of major adverse cardiovascular events (MACEs) in T2DM patients.

In addition to the established benefits of reducing GI by incorporating legumes into bakery products, maintaining stable glycemic control is essential for lowering cardiovascular risk in T2DM patients. A post hoc analysis of the ACCORD study revealed that HbA1c variability, rather than FPG variability, was significantly associated with an increased risk of MACEs in T2DM patients. The study by Sheng et al. (2024) emphasized that greater HbA1c fluctuations, particularly in patients undergoing intensive glucose control therapy, were linked to a 37 % higher risk of MACEs compared to those with lower variability.

Further research has demonstrated that plant polysaccharides, when properly degraded, exhibit enhanced bioactive properties beneficial for functional food applications. Xiaolong et al. (2023) identified multiple degradation methods such as ultrasound, microwave, and enzymatic degradation that effectively reduce the molecular weight and intrinsic viscosity of polysaccharides, improving their functionality in food products. Cereals can significantly vary in their glycemic index (GI) based on factors such as type, processing method and preparation technique. This variability makes cereals a versatile option for developing functional foods, with barley being particularly notable. The health benefits of cereals, including their rich fiber content and low GI in certain varieties, offer substantial potential for creating innovative cereal-based foods and ingredients. These products can be tailored to meet the nutritional needs of specific populations, contributing to improved glycemic control and overall health (Kasote et al., 2021).

## 2 Overview of cereals and legumes

**Fig. 3:**
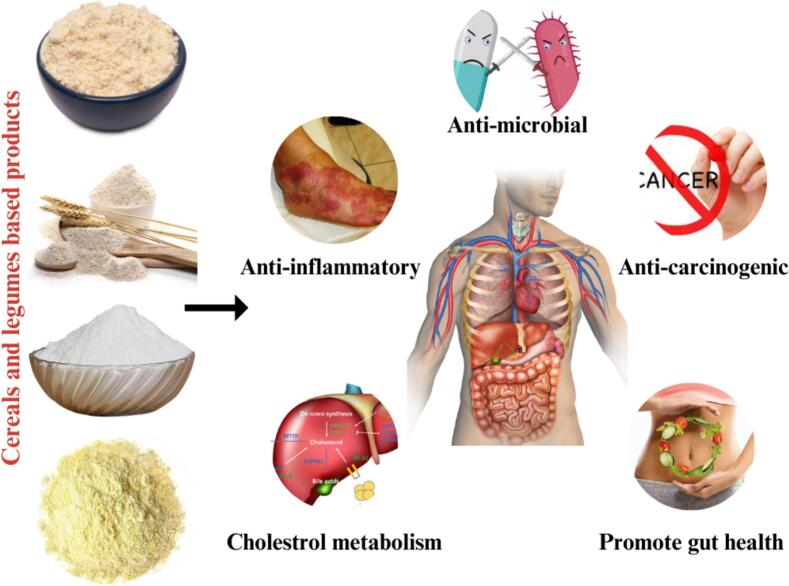
Health benefits of cereal and legume-based food products.

Cereals and legumes play a critical role in the diets of people in low-income developing nations, providing essential nutrients and serving as a key component of a balanced diet (M. Wang et al., 2023). A report from the National Health and Medical Research Council states that cereals like barley, millet, and rye account for over 56 % of human energy and 50 % of protein intake (Otles & Nakilcioglu-Tas, 2022). These grains, along with legumes such as chickpeas and lentils, offer significant health benefits due to their high content of dietary fiber, proteins, vitamins and minerals. Legumes are particularly rich in protein, complex carbohydrates, and essential vitamins like B-complex, making them highly nutritious (Begum et al., 2023). Both cereals and legumes are versatile, consumed in various forms such as whole, refined, or processed products like breakfast cereals (Kaushal & Kumar, 2022). While legumes are known for their nutrient density, they contain anti-nutritional factors like phytates and tannins, which can be mitigated through processing methods such as soaking, cooking, or fermentation to enhance digestibility and nutrient absorption (Samtiya et al., 2020). Certain cereals like barley, millet and rye, as well as legumes like chickpeas, have been identified as particularly beneficial for heart health, blood sugar regulation, and digestive function. Barley, for instance, is notable for its high soluble fiber content and low glycemic index, while chickpeas offer a rich source of folate, iron, and zinc, contributing to better blood sugar regulation and overall digestive health (Obadi & Xu, 2024). The combination of whole cereals and legumes offers a superior nutritional profile due to their complementary properties, cereals being low in lysine, which is abundant in legumes as displayed in Fig. 3. Finger millet and kodo, for example, are powerful sources of antioxidants like phenolics and tannins, which have shown potential in managing glucose levels and oxidative stress in people with type II diabetes (Theodoro et al., 2021). Moreover, studies have linked the consumption of legumes and whole grains with a reduced risk of stomach and colorectal cancer, further underscoring their role in chronic disease prevention (Gaesser, 2020). This Table 1 compiles findings from recent studies on the glycemic impact of various legumes and bakery flours, detailing their nutritional composition, primary bioactive constituents and specific health benefits. By elucidating these parameters, this overview aims to underscore the importance of incorporating legumes and whole grains into dietary regimens to enhance health outcomes and optimize their application in baking practices.

**Table 1:** Studies on the glycemic impact of various cereals and legumes.

**Cereals/** **Legumes**	**Glycemic Index (GI)**	**Nutritional Properties**	**Bioactive Moieties**	**Health Benefits**	**Findings**	**Implications for Baking**	**Reference**
**Chickpeas**	Low (28–32)	High in protein (20 g/100 g), fiber (6 g/100 g) and essential vitamins (B_6_, folate)	Saponins, flavonoids and phenolic acids	Supports weight management and gut health; helps in reducing blood sugar spikes	Low GI promotes better blood sugar regulation; rich in soluble fiber aids in satiety	Ideal for gluten-free baked goods, adding protein and fiber while helping to lower overall GI	(Begum et al., 2023; Xie et al., 2021)
**Barley**	Low (28–35)	Rich in soluble fiber (β-glucan), protein (12 g/100 g), and minerals (selenium, magnesium)	β-glucans, phenolic acids and lignans	Lowers cholesterol levels and aids in heart health; supports digestive function	High soluble fiber content helps regulate blood sugar levels; beneficial for heart health	Suitable for making high-fiber breads and baked products, improving texture and nutritional value	(He et al., 2022; Tosh & Bordenave, 2020)
**Cowpea**	Low (28–32)	High in protein (23 g/100 g), fiber (9 g/100 g) and antioxidants	Polyphenols, flavonoids and tannins	Promotes heart health; aids in reducing blood pressure and improving cholesterol levels	Contributes to improved glycemic control and cardiovascular health	Can be used in flour blends for gluten-free baked goods, enhancing nutritional density	(Bouchenak & Lamri-Senhadji, 2013; Olakanmi et al., 2022)
**Wheat**	Medium (60–70)	Good source of protein (12 g/100 g), carbohydrates and B vitamins	Gliadin, glutenin and phenolic acids	Whole wheat is linked to lower risk of type 2 diabetes and digestive disorders	Whole wheat provides higher fiber content than refined wheat, improving glycemic response	Versatile in various baked products; important to balance with legumes for better GI	(Åberg et al., 2020; Kaushal et al., 2022)
**Millet**	Medium (50–70)	Rich in carbohydrates, fiber (7 g/100 g) and essential minerals (magnesium, phosphorus)	Phytochemicals, phenolic acids and tannins	May improve heart health and aid in blood sugar management; provides sustained energy	Offers energy but has a higher GI compared to other grains; suitable for energy-dense baked goods	Can be combined with lower-GI flours for balanced nutritional profiles in baked goods	(Ghosh et al., 2024; Selladurai et al., 2023)
**Lentils**	Low (21–29)	High in protein (25 g/100 g), fiber (9 g/100 g), iron and potassium	Anthocyanins, flavonoids and tannins	Improves digestive health and supports muscle recovery; beneficial for anemia due to high iron content	Contributes to lower postprandial blood sugar levels; beneficial for managing diabetes	Excellent for gluten-free baking options; enhances texture and nutrient density	(Clarke et al., 2022; Obadi & Xu, 2024)
**Black Beans**	Low (30–40)	High in protein (21 g/100 g), fiber (8 g/100 g) and antioxidants (anthocyanins)	Anthocyanins and flavonoids	Supports gut health and may reduce inflammation; beneficial for heart health due to high fiber content	Provides steady energy release; enhances glycemic control and promotes digestive health	Adds moisture and richness to baked products while improving nutritional profile	(Mullins & Arjmandi, 2021; Perera et al., 2023)
**Pigeon Peas**	Low (35–40)	High in protein (22 g/100 g), fiber (10 g/100 g) and antioxidants (flavonoids)	Flavonoids, tannins and polyphenols	May reduce the risk of chronic diseases; supports heart health and may aid in cancer prevention	Nutrient-rich and associated with improved glycemic control	Adds a unique flavor to baked items; improves nutritional density	(Guaadaoui et al., 2020; Wong et al., 2022)
**Peas**	Low (30–35)	Good source of protein (5 g/100 g), fiber (5 g/100 g) and vitamins (A, C)	Saponins, flavonoids and phenolic acids	Enhances satiety and aids in weight management; supports healthy digestion and immune function	Contributes to lower glycemic responses and offers additional nutrients beneficial for health	Can be used in flour blends to increase protein content and reduce GI	(Kaushal et al., 2022; Tulbek et al., 2024)
**Rye**	Medium (60–70)	High in fiber (14 g/100 g), particularly β-glucan and essential minerals	Lignans and phenolic acids	Promotes digestive health and supports weight management; may help lower cholesterol levels	Good for hearty breads and may improve glycemic control when combined with legumes	Provides a nutty flavor and enhances fiber content in baked goods	(Iversen et al., 2022; Tosh et al., 2020)

### 2.1 Wheat (*Triticum aestivum* L.)

Humans have been using wheat as a main meal since the Late Stone Age (6700 BCE). For more than one-third of the world's population, wheat is among the most important staple food crop, contributing more calories and protein to the diet than any other cereal grain (Xynias et al., 2020). A nutritious and adaptable grain, wheat can be used in a large range of dishes. It is considered as a good source of minerals, B-group vitamins, proteins and dietary fiber. Wheat persists as a wholesome grain that is useful to the body even though environmental influences can alter its nutritional makeup, especially in its important bran layer where most minerals and vitamins are concentrated. During baking, almost 10 % of the lysine in wheat flour is lost, which is problematic as it is the first limiting amino acid. Bread, biscuits, candies, noodles and wheat gluten (seitan) are all derived from wheat flour. It is also used for cosmetics, ethanol manufacturing, animal feed, beer brewing, meat alternatives and wheat straw composites. Wheat germ and bran are high in dietary fiber, which can help prevent and treat some digestive issues. Eleven distinct wheat varieties, including red, white and durum wheat, were utilized to test the antioxidant activity and phytochemical composition of milled grain. One of the main benefits of wheat above other temperate crops is the unique qualities of its flour dough, which can be used to make a wide variety of baked items, among other things. These properties depend on how the storage proteins in the grain interact and are structured. These proteins comprise the gluten component (Awulachew, 2020). Wheat is rich in lutein, the most abundant carotenoid found in it, with the germ and bran fractions containing higher levels of carotenoids and antioxidant activity compared to the endosperm. Similar to zeaxanthin, lutein is essential for human skin and eye health (P. Kumar et al., 2011; Rawat et al., 2023). Additionally, processing methods like milling, extrusion and cooking can further impact wheat's glycemic response as shown in Table 2.

**Table 2:** Effects of processing techniques on the glycemic index of cereal and legume-based products.

**Processing Techniques**	**Effect on Glycemic Index**	**Explanation**	**Food Types Affected**	**Reference**
**Baking**	Increase or decrease in GI depending on formulation and ingredients	Baking can cause starch gelatinization, which affects digestibility and GI. The addition of fiber or legume flour tends to lower GI.	Cereal-based (bread, cakes, cookies), legume-based (baked goods with chickpea, lentil flour)	(Tagliasco, 2024)
**Fermentation**	Significant reduction in GI	Fermentation can reduce the available carbohydrates by breaking down starch and sugars, leading to a lower GI.	Cereal-based (sourdough bread), legume-based (fermented soy, tempeh)	(Demirkesen-Bicak et al., 2021)
**Cooking (Boiling)**	Generally, lowers GI, especially in legumes	Boiling increases the digestibility of starches and legumes, leading to a slower release of glucose and a lower GI.	Legume-based (lentils, chickpeas, beans), some cereals (oats, barley)	(H. Meng et al., 2017)
**Milling (Refining)**	Increase in GI due to removal of fiber and bran	Milling removes the outer layers (bran and germ) of grains, which lowers fiber content and increases GI.	Cereal-based (white bread, pasta)	(Vega-López et al., 2018)
**Sprouting**	Decrease in GI	Sprouting enhances fiber content and reduces starch digestibility, resulting in a lower GI.	Cereal-based (sprouted wheat, barley), legume-based (sprouted lentils, beans)	(Lemmens et al., 2019)
**Drying**	Variable effect depending on method (sun-dried vs. industrial drying)	Drying methods impact starch crystallinity and sugar content, with industrial drying leading to higher GI.	Legume-based (dry beans, lentils), some cereals (corn, rice)	(Zhang et al., 2021)

Dietary fiber, proteins, minerals and water-soluble vitamins are all found in abundance in wheat. The bran (13–17 %), endosperm (80–85 %) and germ (2–4 %) of the wheat grain are the three sections that hold all of the essential nutrients. Wheat kernels are primarily composed of carbohydrates, minerals, protein, water, fat and crude fiber. Additionally, they are a rich source of essential minerals, including zinc, magnesium, phosphorus, manganese, selenium, copper, iron and potassium. Furthermore, wheat grain kernels contain bioactive compounds that contribute to their nutritional value. These components collectively enhance the functional properties of wheat, making it a valuable crop in human nutrition and health. Phenolic acids are abundantly dispersed throughout grains including the pericarp, testa and aleurone. Wheat contains a range of phenolic acids, including chlorogenic acid, caffeic acid, ferulic acid, sinapic acid and p-coumaric acid. These compounds are mainly present in bound forms, with phenolic acids comprising about 85 % in wheat, 75 % in maize and 62 % in rice (Arshad et al., 2017). Iwe and Egwuekwe (2010) used combinations of wheat and *Xanthosoma sagitifolum* and wheat with *Colocasia esculenta* flour to make the biscuits. In the ratios of 25:75, 50:50, 100:0 and 0:100, researchers blended wheat flour with the flour of each cocoyam species. The panelists found the products to be satisfactory based on the findings of their sensory inspection. Other than the 25:75 wheat-*Xanthosoma* flour biscuit, the biscuit composed solely of wheat flour had a much better appearance (*P* < 0.05). The taste of the biscuits prepared with 100 % wheat flour was significantly better than those made with 100 % *Xanthosoma* and 25:75 wheat-*Colocasia* flour.

### 2.2 Fractions of wheat flour

Wheat flour consists of various distinct fractions, each with unique properties and specific applications, typically obtained during the milling process. These fractions include components such as the bran, germ, and endosperm, which are separated and processed to create different types of flour. Among these, all-purpose flour, made from the finely milled endosperm, is the most versatile and widely used in baking due to its balanced protein content. It is suitable for preparing a wide range of baked goods, including cakes, cookies, pastries and bread (Atwell & Finnie, 2016).

#### 2.2.1 All-purpose flour

All-purpose flour is the most commonly used type of flour for baking, as it can be used to prepare a wide variety of baked goods, including cakes, pastries, cookies, and yeast breads. Its moderate protein content, typically ranging between 8 % and 11 %, is a result of blending soft and hard wheat cultivars. This flour is derived from the endosperm, which is finely ground after being separated from the germ and bran during the milling process, making it a versatile ingredient for both home and commercial baking. (P. Kumar et al., 2011). All-purpose flour is produced from a blend of both soft and hard wheat, which is why it is referred to as “all-purpose.” It is a staple ingredient in the food industry, particularly in the production of a wide range of baked goods. However, despite its versatility, there are several drawbacks to its use that can impact both consumer health and the overall quality of the final product. One key concern is the refining process, which removes the bran and germ, stripping the flour of essential nutrients like fiber, vitamins and minerals. This can result in a product that is lower in nutritional value and may contribute to health issues, such as insulin resistance and weight gain, when consumed in excess. Additionally, its high glycemic index can lead to rapid blood sugar spikes, which is another factor to consider when using all-purpose flour in baking. (Bressiani et al., 2017). White flour has several drawbacks when used in baked products. During the refining process, all-purpose flour undergoes significant nutrient loss. The bran and germ, which are rich sources of fiber, vitamins, minerals, and phytochemicals, are removed during the high level of refinement. As a result, this process strips the flour of essential nutrients, leading to potential deficiencies in vital components such as B vitamins, vitamin E, magnesium, and fiber. This nutrient loss can contribute to health issues when white flour is a major part of the diet, as it lacks the nutritional benefits found in whole grain alternatives (Charles et al., 2022). This results in a product with significantly fewer essential nutrients, such as vitamins B and E, magnesium, and iron. The removal of these key elements during refinement reduces the overall nutritional value of white flour, making it less beneficial for maintaining a balanced and healthy diet compared to whole grain alternatives.

Choosing whole grain flours and products can help maintain a balanced diet rich in essential nutrients, promoting overall well-being and health. Whole grain flours retain the bran, germ, and endosperm, providing higher levels of fiber, vitamins, minerals and antioxidants. These nutrients support better digestion, improved heart health, and more stable blood sugar levels, making whole grains a healthier option compared to refined flours (Anushka & Sianne, 2022). The high glycemic index of processed all-purpose flour causes rapid variations in blood sugar levels. Insulin resistance, weight gain and a higher chance of type II diabetes may arise through it. White flour-based products are generally high in calories and low in nutrients, leading to obesity. Because of the lack of dietary fiber, these items tend to be less filling, which might contribute to over eating (Jerome et al., 2019). To reduce these adverse effects, choose low- glycemic index foods, include fiber and protein into food and eat a balanced diet that encourages stable blood sugar levels. By adopting these modifications, people can improve their overall health and reduce their risk of developing chronic illnesses linked to food high in glycemic index (Pruett et al., 2023). All-purpose flour has been referred to as the “glue of the gut” due to its potential to clog the digestive system, inhibit proper digestion, and contribute to a slower metabolism. Its low fiber content, combined with gluten exposure, can negatively affect gut health and disrupt the balance of gut microbiota. Regular consumption of all-purpose flour may increase the risk of digestive issues and systemic inflammation. To support digestive health and reduce the risk of these problems, individuals should opt for whole grains, manage gluten sensitivity, incorporate probiotic and prebiotic foods into their diet, and maintain a healthy lifestyle. These changes can promote better digestion and overall well-being (Salis et al., 2021). Further processing techniques, such as bleaching or the addition of preservatives, can also affect the glycemic index of flour by altering its structure. These modifications can make the carbohydrates in the flour more easily digestible, leading to faster spikes in blood glucose levels. As shown in Table 2, these processing methods can increase the glycemic index, further reducing the nutritional value of the flour and its overall health benefits. In addition to all-purpose flour, consider using alternative flours such as barley, which is high in fiber, and chickpea, which is rich in protein. These and other whole grain flours can create healthier, more nutritious, and flavorful baked items. Barley flour helps lower the glycemic index of baked goods and supports digestive health, while chickpea flour adds protein and essential nutrients. Incorporating these flours into your baking can enhance both the taste and the nutritional profile of the final products (Alrayyes, 2018).

### 2.3 Barley (Hordeum vulgare L.)

The interactions and structure of barley's storage proteins, which include gluten, play a key role in determining its functional characteristics. Barley is cultivated globally, thriving in a variety of climates, from temperate regions in the summer to areas with temperate and subtropical conditions during the winter. Its adaptability makes it a versatile crop for various food and industrial uses (Djanaguiraman et al., 2020). Barley was probably first utilized as animal fodder and now it is used in malt production. Later, as wheat became more prevalent in human consumption, barley became an essential crop for the production of feed as well as malt manufacturing. Throughout history, it has been the primary food supply for various cultures, particularly in North Africa and Asia (Abebaw, 2021). According to Geng et al. (2022) gaining momentum in the food industry owing to its fiber content especially β-glucan lowers the risk of blood cholesterol, glycemic index and lessen the risk of cardiovascular illness. Biscuits, pasta and bread manufactured from barley flour are not yet widely available, but they are gaining increasing importance in the manufacturing industry. But it is appropriate for consumption by humans, barley must go through numerous stages of processing (baking, frying and extrusion), having a substantial impact on its composition and physicochemical attributes (Narwal et al., 2020). Dietary fiber, particularly soluble fiber, functions as a prebiotic by being fermented by gut microbiota to produce short-chain fatty acids (SCFAs) such as acetate, propionate, and butyrate. These SCFAs are vital for maintaining a healthy gut environment (Ashaolu et al., 2021). Butyrate, in particular, serves as a primary energy source for colonocytes (intestinal cells), supports cell differentiation, and helps maintain the intestinal barrier. Fiber also promotes the growth of beneficial gut bacteria, including *Bifidobacteria* and *Lactobacilli*, while inhibiting the growth of harmful microorganisms. This balanced microbial environment is essential for gut health and plays a crucial role in preventing conditions such as inflammatory bowel disease (IBD) and irritable bowel syndrome (IBS) (H. Wang et al., 2022).

**Fig. 4:**
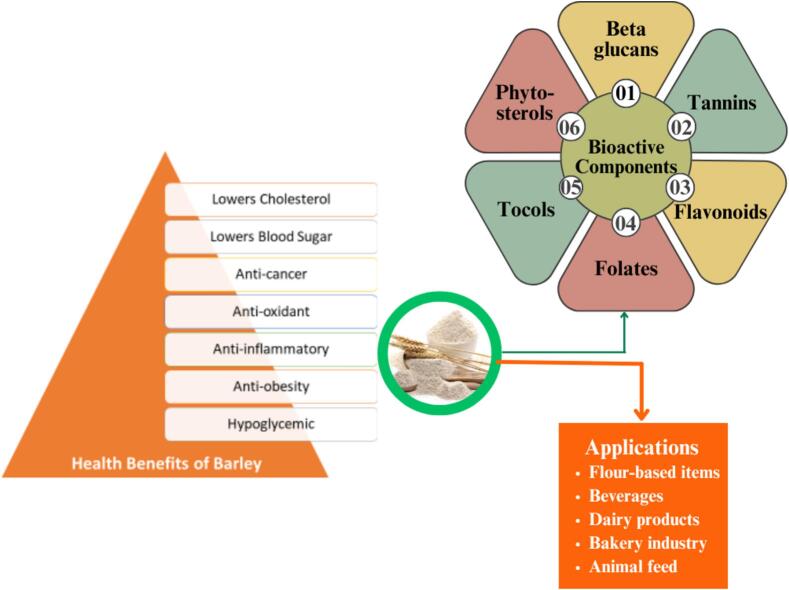
Schematic representation of barley bioactive components, health benefits and applications.

Barley grain is mostly made up of carbohydrates (such as starch, sugars and dietary fiber), proteins (amino acids), fat (fatty acids), vitamins and minerals (Geng et al., 2022). Barley is recognized as a useful ingredient in the creation of goods that improve human health due to its chemical makeup. Fig. 4 presents a schematic representation of the bioactive components found in barley, highlighting their associated health benefits and various applications. This Fig. 4 illustrates the wide-ranging roles that barley plays in promoting health, from supporting digestive function to regulating blood sugar levels, and emphasizes its versatility in a variety of consumer products, including baked goods, beverages, and functional foods. Through these applications, barley contributes significantly to overall wellness and disease prevention. In recent years, numerous scientific expectations demonstrated that consuming barley food products has favorable benefits on glucose metabolism (Fuse et al., 2020). Barley often has a lower glycemic index (GI) than other cereals, ranging from 25 to 85 for rice, 52 to 75 for wheat and 46 to 80 for maize. However, preparing a dish or combining it with other foods can alter its glycemic index value (Lal et al., 2021). Research has demonstrated that barley grains have a significant ability to make products with very low glycemic index, notably amylose-only barley grains that contain 99 % amylose starches. Barley foods reduce blood sugar due to their β-glucan content and amylose/amylopectin ratio. The glycemic response to foods containing barley is generally determined by combining these two factors. The findings indicate that a diet comprised of wild barley or genetically modified amylose-only barley grain has the ability to reduce and anti-diabetic consequences for post-prandial blood glucose response (Sagnelli et al., 2018).

Punia Bangar et al. (2022) emphasized on utilizing twin-screw extrusion technology to enrich barley flour with various health-promoting ingredients. It examined the way barley flour's resistant starch, glycemic index and antioxidant qualities were affected by extrusion processing, showing promise for the creation of high-fiber, ready-to-eat snacks with improved nutritional profiles. Improved resistant starch, a lower glycemic index and increased antioxidant activity were all demonstrated by the results. Aly et al. (2024) investigated the anti-obesity effects of incorporating whole barley flour into bread. This study contributes to the increasing amount of data demonstrating the health advantages of using barley flour in food products. The impact of replacing chickpea flour in flatbread with barley flour was evaluated by Mansoor et al. (2021), who demonstrated that the inclusion of barley flour improved the flatbread's texture, lowered its glycemic index and improved its sensory qualities. The research highlights how barley flour can be used as a functional ingredient to improve food products' nutritional value and acceptability by consumers. However, the glycemic index of barley can be significantly influenced by various processing techniques. For instance, refining or milling barley into flour increases its glycemic index by disrupting the fiber and starch structure, making it quicker to digest. This leads to faster spikes in blood sugar levels, as indicated in Table 2. Thus, while barley offers numerous health benefits, the way it is processed can affect its impact on blood glucose regulation.

### 2.4 Chickpea (Cicer arietinum L.)

**Fig. 5:**
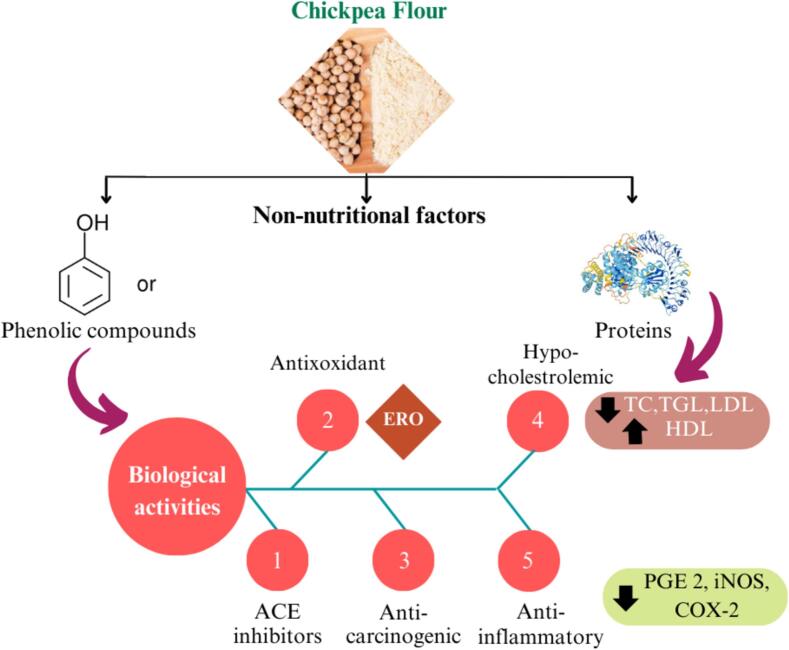
Chickpeas (*Cicer arietinum L.*): bioactive components and biological activity in relation to human health.

The *Fabaceae* family includes the annual plant, chickpea (*Cicer arietinum*), which is predominantly grown in temperate to semiarid regions including parts of Asia, Europe, Australia and North America. Its adaptability to various climates makes chickpea a valuable crop for both nutritional and agricultural purposes in these regions (Rani et al., 2020). Approximately 66 % of the world's chickpea production comes from India, making it the leading global producer. Turkey ranks second, contributing 7.6 %, followed by Pakistan at 7 % and Iran at 3.5 %. In contrast, the United States and Canada contribute minimally to global chickpea production, with 1.6 % and less than 1 %, respectively (Koul et al., 2022). The two most common types of chickpeas are Kabuli and Desi. The tiny, black grains of Desi chickpea have a ridged surface. Semiarid areas are the main places where the Desi variety is grown (Jameel et al., 2021; Zetochová et al., 2022). The differences in macro and micronutrient composition between Kabuli and Desi chickpeas arise from several factors (Xiao et al., 2023). Their distinct genetic backgrounds influence nutrient profiles, while environmental conditions, such as soil type and climate, affect nutrient uptake. Agricultural practices, including fertilization and irrigation, also play a role. Additionally, the thinner seed coat of Kabuli chickpeas may improve nutrient absorption compared to the thicker seed coat of Desi chickpeas, potentially influencing nutrient release during cooking. Different culinary applications and processing methods can further alter their nutrient composition(Jayalakshmi & Kumar, 2023). In comparison, chickpea flour contains higher levels of protein, ash, fat and fiber, along with significant biological effects, as demonstrated in Fig. 5. Though they are rich in amino acids, chickpeas lack certain of them. Aspartic acid and arginine are the limiting amino acids in chickpea. However, consuming pulses along with grains might make up for the deficiency of these amino acids and meet a person's dietary requirements (S. Kumar et al., 2022).

Although the glycemic effects of hummus in vivo have not been extensively studied, chickpeas have a naturally low glycemic index. Hummus, a nutritious and flavorful Middle Eastern spread made from cooked and mashed chickpeas, has shown promising effects on blood glucose levels. In a study comparing hummus to white bread, ten healthy adults experienced postprandial glucose responses that were four times lower after consuming hummus. After 45 min, participants' blood glucose levels were significantly lower when they consumed hummus with 25 g of readily available carbohydrates compared to those who consumed white bread with the same carbohydrate content. This suggests that hummus may help mitigate the adverse effects of high-glycemic index foods, as it resulted in a postprandial glucose level that was four times lower than that of white bread without impacting insulin levels (Augustin et al., 2015). Another study evaluated the substitution of 10 % to 40 % chickpea flour for wheat flour in biscuit production. The findings showed that incorporating chickpea flour into wheat flour increased the water solubility of the mixture and reduced its swelling capacity. Higher levels of chickpea flour incorporation also raised the content of slowly digested and resistant starch in the biscuits, increased the gelatinization onset temperature, and reduced the gelatinization energy and viscosity. These changes contribute to improved nutritional and textural properties in the final product, making the biscuits healthier with better starch digestibility (Lu et al., 2022).

The potential of chickpea flour to improve the nutritional profile of baked goods was highlighted by Rizzello et al. (2014) as they investigated the use of sourdough fermentation and a combination of lentil, chickpea, wheat and bean flours to boost the nutritional features of bread. Using a combination of wheat-legume sourdough and regular wheat sourdough, two bread slices with 15 % (*w*/w) legume flour made from chickpeas, lentils and beans were made. These sourdoughs had higher levels of total free amino acids compared to wheat yeasted bread. Wheat-legume sourdough had the strongest phytase and antioxidant properties. The addition of bean flours decreased the in vitro digestibility of proteins as compared to wheat yeasted bread. However, dough fermentation with wheat- legume sourdough promoted an increase in in vitro protein digestibility. According to the quantities of resistant starch, carbohydrates and dietary fibers, wheat-legume sourdough and wheat sourdough had lower hydrolysis index levels than wheat yeasted bread. Various processing techniques, such as milling, cooking, and fermentation, significantly influence the glycemic index (GI) of cereal and legume-based products by altering carbohydrate structure and digestion rate. These methods can either raise or lower the GI depending on factors like particle size, starch gelatinization and fiber breakdown. Understanding the impact of these techniques is crucial for designing foods with a lower GI, aiding in blood sugar management and enhancing the nutritional quality of these staple foods, as demonstrated in Table 2.

Mansouri et al. (2024) investigates the effects of non-soy legume consumption on inflammatory biomarkers and adiponectin levels in overweight and obese adults. The study synthesizes data from multiple randomized controlled trials to assess how legumes such as lentils, chickpeas and beans impact key biomarkers of inflammation and adiponectin, a hormone involved in regulating metabolism and fat storage. The findings suggest that regular consumption of non-soy legumes significantly reduces inflammatory markers like C-reactive protein (CRP) and interleukins, while also increasing adiponectin levels, which are associated with improved insulin sensitivity and fat metabolism. These results highlight the potential of non-soy legumes as a dietary intervention for managing inflammation and metabolic health in individuals with obesity, supporting the growing evidence for legumes' role in chronic disease prevention.

### 2.5 Environmental impact and sustainability of promoting legume and cereal-based diets

Promoting legume and cereal based diets is increasingly recognized as a sustainable approach to addressing the environmental challenges associated with food production. Legumes, such as beans, lentils and chickpeas, have a notably low environmental footprint, requiring fewer natural resources like water, land, and fertilizers compared to animal-based protein sources. They also improve soil health through nitrogen fixation, reducing the need for synthetic inputs (Cusworth et al., 2021). Similarly, cereal crops like oats, barley and wheat offer resource-efficient alternatives, with lower greenhouse gas emissions and water usage than livestock farming. Transitioning to more plant-based diets, centered on legumes and cereals, can help mitigate climate change, conserve biodiversity and promote more resilient and sustainable food systems globally. This shift aligns with modern sustainability goals and provides a critical pathway for reducing the ecological impact of food production while supporting global food security (Boukid, 2024).

In addition to their environmental benefits, legumes and cereals also contribute to sustainable agricultural practices by supporting crop rotation and diversification. These crops typically require fewer external inputs, making them a cost-effective choice for small-scale farmers in developing regions. Furthermore, promoting these foods could foster local food sovereignty by reducing dependence on imported animal products, thus strengthening local economies and food systems (S. Kumar et al., 2020). As consumers increasingly prioritize sustainability, the demand for legume and cereal based products is likely to grow, driving further innovation in plant-based food production and processing technologies. Supporting this shift in dietary habits will be crucial in meeting the food security and environmental goals set by global initiatives like the United Nations Sustainable Development Goals (SDGs), especially those focused on hunger, health and climate action (Mugambiwa & Tirivangasi, 2017).

### 2.6 Impact of cereal-based products on blood sugar levels

**Fig. 6:**
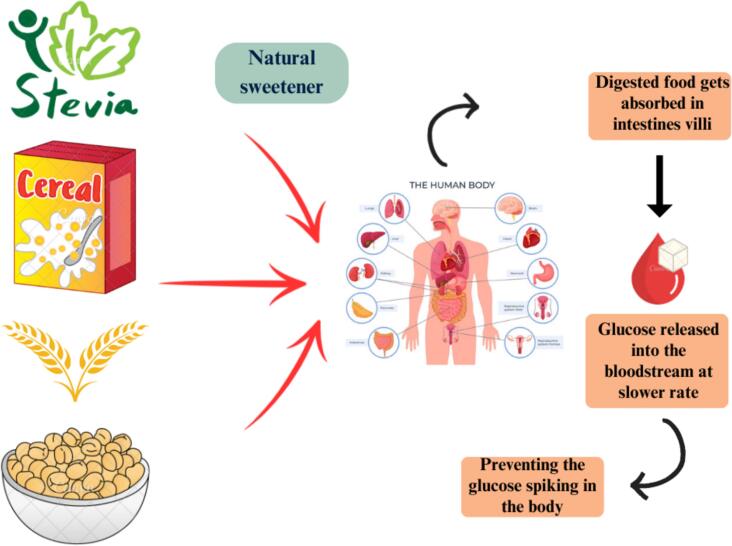
Cereals and the metabolism of low glycemic index biscuits.

The glycemic index (GI) measures how much a carbohydrate-containing food raises blood glucose levels compared to a reference food, such as glucose or white bread (Kim et al., 2019). It is calculated based on the incremental area under the blood glucose curve for a test food portion. A GI score of 70 or higher is considered high, 56–69 is moderate and 55 or lower is low (Anh et al., 2021). A glycemic index rating of 70 or above is considered high, 56–69 is considered medium and 55 or lower is considered moderate. The glycemic index of a food is influenced by several factors (Lal et al., 2021). Cooking and processing methods typically increase the glycemic index, while whole grains, beans and lentils, with their intact fibrous coats, act as a physical barrier that slows digestion as shown in Fig. 6. The type of starch in the food also plays a role, as does portion size larger servings have a greater impact on blood glucose. When different foods are consumed together, as is common at mealtimes, the overall glycemic index is determined by the combined quantities of all the items. Additionally, the fat content in a food product can lower its glycemic index (Silva et al., 2020; M. Singh et al., 2021). It can be challenging to determine the glycemic index of a specific item or food that as a consequence. Many of these problems have been described in a study along with recommended techniques (Wee & Henry, 2020). When foods are consumed together, their combined GI is determined by the mix of items. To provide a more accurate reflection of a food's impact on blood sugar, the concept of glycemic load (GL) was developed, which accounts for both the GI and the quantity of carbohydrates in a typical serving (Barclay et al., 2021; Jenkins et al., 2021).

### 2.7 Glycemic load and its effect on satiety

The glycemic load (GL) is a dietary tool developed to help individuals manage blood sugar and insulin levels, while also reducing cholesterol levels associated with cardiovascular disease (Marbini et al., 2021). Low-glycemic diets have been shown to be beneficial for managing diabetes and cholesterol, according to numerous meta-analyses (Venn & Green, 2007; Yau et al., 2020). Adhering to a glycemic index (GI) or GL-based diet may lower blood glucose and insulin levels, reducing the risk of coronary heart disease and type II diabetes (Liang et al., 2022). The effect of a food's glycemic response on weight management and satiety was examined by Bornet et al. (2007). The evaluation included both short-term trials (one day or less) with meals or liquid carbohydrates (fructose or glucose) and longer-term studies (two weeks to six months). Out of 26 selected studies, most focused on cereal-based foods like bread, spaghetti and muffins. Despite some unreliable results, 16 studies found that low glycemic index foods promote greater satiety than high glycemic diets. Overall, the research indicates that low glycemic foods provide longer-lasting fullness compared to their high glycemic counterparts.

A randomized controlled trial compared the effects of two energy-restricted regimens: one with high glycemic load and the other with low glycemic load (Che et al., 2021). During the first six months, participants followed personalized diets designed to minimize factors like palatability, variety and support. Over three months, they were assessed for satisfaction with food quantity and variety, as well as cravings for non-study foods. Satisfaction dropped significantly in the high-glycemic load group but remained stable in the low-glycemic load group. Both groups reduced calorie intake and lost weight and body fat, but no significant differences were observed between the high- and low-glycemic load groups (Aisbitt et al., 2008; Das et al., 2007). Research suggests that while glycemic index-controlled diets may enhance short-term satiety, they do not lead to greater long-term weight loss compared to other energy-controlled diets. Low-glycemic load meals are typically high in fiber, which could be a confounding factor in these findings. Fiber is known to promote satiety by increasing the volume of food and slowing nutrient absorption in the gut. Therefore, increasing fiber content in cereals may be more effective in enhancing satiety than simply focusing on lowering the glycemic index (Mecha et al., 2021).

## 3 Use of Cereals in Baking Industry

Bakery products are widely consumed by people of all ages around the world due to their delicious flavor and easy digestibility. Items such as cookies, biscuits, bread, tortillas, cakes, muffins, wafers, rolls and pies are now readily available, increasing accessibility for consumers (Bauer et al., 2022). In response to these trends, bakeries are increasingly considering the use of Process Analytical Technology (PAT), developed by the U.S. Food and Drug Administration, to enhance the safety, quality, and efficiency of baked goods production. The bread market was valued at over US$ 7.22 billion in 2018, with a 2.5 % increase in retail sales (Dholakia et al., 2018). In the bakery sector, where a large volume of products is produced daily, there is a strong demand for real-time tools to assess quality. The global bakery market is projected to reach $20.99 billion by 2023. Recently, efforts have been made to enhance production and quality in Pakistani bakeries through the adoption of innovative baking equipment and techniques (Ijaz & Mateen, 2022). Cereals play a crucial role in baking, contributing to the creation of a wide range of tasty and nutritious products (Biljwan et al., 2019).

In Pakistan, bread production, both homemade and industrial, relies heavily on baker's yeast, with only a small portion using sourdough fermentation (Rahman et al., 2022). Wheat is the primary grain used in Pakistan's baking industry. The performance of wheat varieties such as NARC 2009, Galaxy 2013, NARC 2011, Zincol 2016, Pakistan 2013, and Borlaug 2016 has been assessed for producing high-quality pan bread (Asim et al., 2018). However, limitations in protein content in certain wheat varieties, such as Zincol 2016, may affect their suitability for baking (Ahsin et al., 2023). Additionally, the use of indigenous ingredients like whole wheat, local grains, and pulses is gaining traction in the baking industry, especially due to recent initiatives promoting healthier diets (Mbachu et al., 2023). Cereals such as wheat, maize, and rice are essential to the baking industry because of their abundance, variety, and nutritional value (Senarathna et al., 2024). Moreover, the rising demand for gluten-free products has driven the development of innovative baking techniques and formulations using alternative grains and starches, providing solutions for individuals with gluten intolerance or celiac disease (Gómez, 2022).

To enhance the applicability of the review in real-world settings, practical recommendations for consumers and public health practitioners are essential. For instance, suggesting simple ways to incorporate cereal and legume-based bakery products such as oat flour pancakes, chickpea bread or barley muffins, into daily meals can help improve glycemic control and overall health (Siddiqui et al., 2022). Public health campaigns could promote these alternatives in place of refined wheat-based products, offering tips on how to easily substitute legume and whole grain flours in traditional recipes. Additionally, meal planning examples for diabetic patients, such as including a slice of lentil-based bread with a vegetable-packed salad or pairing quinoa bread with lean protein, can help individuals make informed food choices that support better blood sugar management and prevent complications associated with diabetes.

### 3.1 Biscuits and their relation to low glycemic index

Biscuits are minimal baked goods made up of primarily flour, sugar and fat (Mamat & Hill, 2018). Biscuits vary from other baked products such as cakes and bread in that they contain less moisture. Its moisture level is usually below 4 %, hence it has an extended shelf life possibly six months or more (Ma et al., 2020). In general, there are two varieties of biscuit dough: firm and soft. Semi-sweet biscuits are made from hard, developed dough. Hard dough contains high level of moisture and less fat and sugar (Kouhsari et al., 2022). Popular food items are effective carriers for nutrient assimilation, making them appealing to a burgeoning and increasingly demanding market for health condition management. Among these items, biscuits show interest as an enhanced food for meeting nutritional demands or preventing diet-related ailments (Aderibigbe et al., 2022). Biscuits provide numerous options for the treatment of chronic illnesses. They are commonly eaten as snacks or as a supplement to other foodstuffs. They come in a variety of forms and flavors, have a prolonged lifespan and are convenient (Ghnimi et al., 2023). As a result, biscuit production and consumption have expanded dramatically over the world. Within the fast- moving consumer products sector, one of the biggest industries is biscuits (Sen, 2021).

**Fig. 7:**
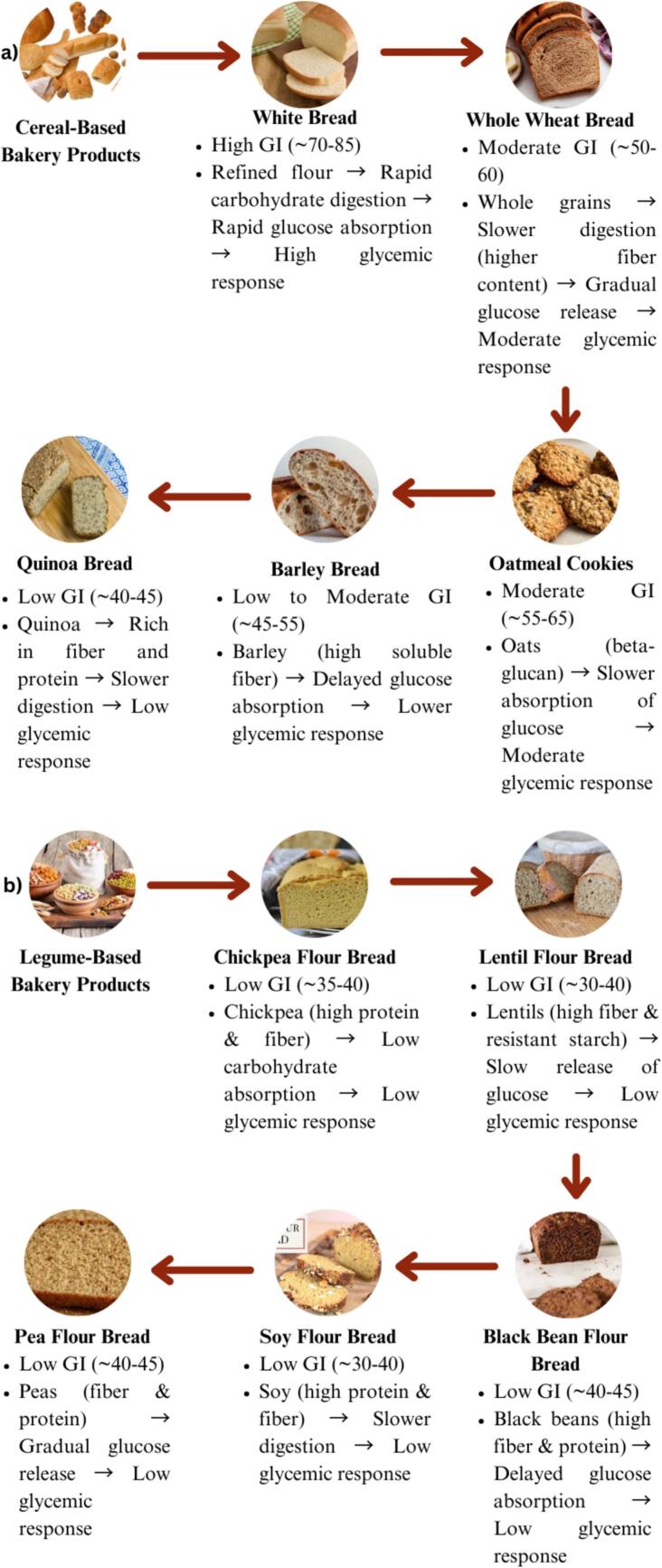
Glycemic index of different legume and cereal-based bakery products.

Relative to the huge variety of biscuits available today, the number of investigations enhanced biscuits and their application in clinical studies is quite small (Xu et al., 2020). Kabir et al. (1998) discovered that when starches with varying amylose-to-amylopectin ratios (32:68 in mung beans and 0.5:99.5 % in waxy cornstarch) were incorporated into a mixed meal, the food with the higher amylopectin content (waxy cornstarch) exhibited a higher glycemic index compared to the lower amylopectin starch. The study found that in both normal and diabetic rats, long-term replacement of a high glycemic index starch with a low glycemic index starch in a mixed diet increased insulin-stimulated glucose oxidation, reduced glucose incorporation into total lipids and decreased epididymal adipocyte diameter. Thus, the inclusion of such starch in the diet has significant metabolic effects at the cellular level in both diabetic and normal rats. Additionally, the inclusion of components like tiger nut and coconut flour can affect the anticipated glycemic value of biscuits (Ogunka-Nnoka et al., 2020; Pathirana et al., 2020). In comparison to wheat flour (48.18 %), the tiger nut biscuit had the highest protein digestibility (52.53 %) and the other biscuit had the lowest (39.44 %), according to the results of the in-vitro protein digestibility of the composite biscuit. The composite biscuits had the highest starch digestibility, measuring 99.45 ± 2.32 % and 89.99 ± 1.67 %, respectively. The biscuits had the greatest hydrolysis index, measuring 138.85 and 113.79 %, respectively. This led to higher predicted glycemic index values for composite biscuits, respectively of 115.93 and 102.17 %. These clinical studies on cereal and legume-based bakery products have highlighted their potential in managing glycemic response and chronic diseases. These studies often compare the GI of traditional wheat-based bakery products with those made from legumes (e.g., chickpeas, lentils) or whole grains like barley and oats as shown in Table 3. The Fig. 7 highlights the GI of various bakery products made from legumes (e.g., chickpeas, lentils) and whole grains (e.g., barley, oats).

**Table 3:** Clinical studies on cereal and legume-based bakery products and their glycemic impact and potential for chronic disease management.

**Product Type**	**Clinical Studies**	**Findings**	**Glycemic Impact**	**Potential for Chronic Disease Management**	**Reference**
Legume-based bread (chickpea)	Glycemic response in type II diabetes	Chickpea bread improved postprandial glucose levels	Lower glycemic response compared to wheat bread	Helps in diabetes management	(Ferreira, 2020)
Barley-based biscuits	Impact on cholesterol and glycemic control	Barley biscuits reduced total cholesterol and glycemic index	Low glycemic index, improved cholesterol profile	Reduces cardiovascular risk, aids in blood sugar control	(Lazaridou et al., 2022)
Millet-based muffins	Glycemic response in healthy individuals	Millet muffins showed significantly lower post-meal glucose spikes	Moderate glycemic reduction compared to refined wheat muffins	Supports blood sugar management and weight control	(Almaski et al., 2023)
Lentil-based bread	Diabetes prevention and management	Lentil bread improved glycemic control and insulin sensitivity	Low GI, beneficial for glycemic control in diabetic patients	Could reduce the risk of type II diabetes	(Moravek, 2018)
Sorghum-based bakery products	Effects on glycemic control and obesity	Sorghum products reduced postprandial glucose and improved satiety	Low glycemic index, slower glucose absorption	Assists in managing obesity and type II diabetes	(Girard & Awika, 2018)

**Flour Type**	**Sustainability Considerations**	**Accessibility Considerations**	**Regional Availability**	**Key Limitations**	**Table:** **Reference**
Barley Flour	Requires less water than wheat and is more drought-resistant	Available in regions with strong barley farming traditions	Widely grown in temperate regions, especially Europe and North America	Limited in tropical and subtropical climates	(Olugbire et al., 2021)
Chickpea Flour	Legumes like chickpeas enrich soil nitrogen, reducing fertilizer use	Widely available in the Middle East, South Asia and Mediterranean regions	Predominantly produced in India, Turkey, and parts of Africa	Limited processing infrastructure in non-producing regions	(Binou et al., 2022)
Wheat Flour	Large water usage and reliance on chemical inputs, contributing to environmental impact	Cheapest and most widely available flour globally	Available worldwide, especially in industrialized countries	Environmental costs from monoculture practices	(Erenstein et al., 2022)
All-Purpose Flour	Similar environmental concerns as wheat flour but with lower nutritional content	Cheapest and most widely used flour globally, especially in industrialized nations	Available in nearly all regions, especially in developed economies	Lacks the nutritional benefits of whole grains and alternative flours	(Burt et al., 2020)

**Table: 4** Regional sustainability and accessibility of alternative flours.

## 4 Modulation of Metabolic Pathways by Cereal and Legume-Based Products

**Fig. 8:**
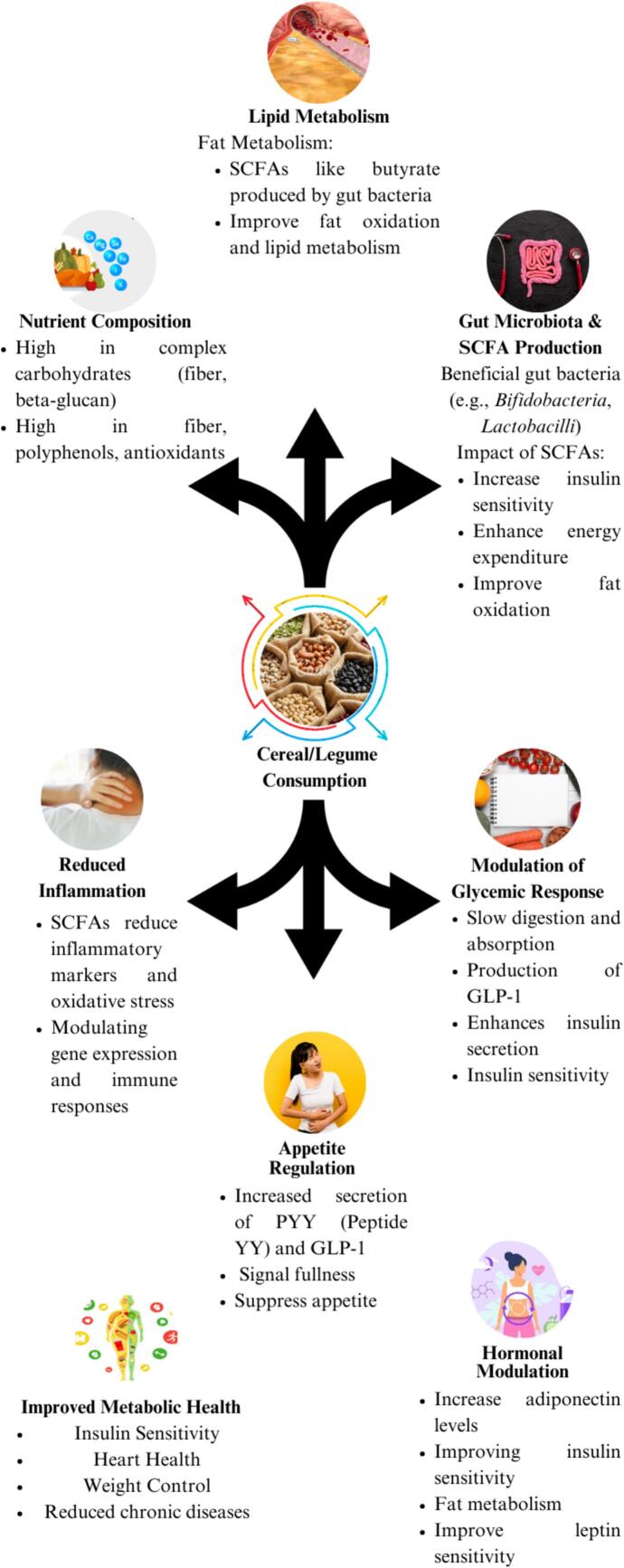
Modulation of metabolic pathways by cereal and legume-based products.

Cereal and legume-based products play a crucial role in supporting key metabolic pathways, contributing to improved metabolic health. These foods are rich in fiber, protein, and bioactive compounds that positively influence glucose metabolism, lipid profiles, and inflammation. Whole grains like oats, barley, and quinoa provide soluble fiber such as beta-glucan, which slows carbohydrate absorption and helps maintain steady blood glucose levels(Allai et al., 2022). Legumes, including chickpeas, lentils and beans, have a low glycemic index, high protein content and resistant starch, which together help regulate blood sugar, reduce insulin resistance and promote satiety. Both cereals and legumes also support gut health by fostering beneficial gut microbiota that produce short-chain fatty acids (SCFAs) such as butyrate, known for their anti-inflammatory effects and their role in enhancing insulin sensitivity (Yao et al., 2020). The consumption of these foods provides a holistic approach to managing chronic conditions like type II diabetes, obesity and cardiovascular diseases, promoting overall metabolic balance, as illustrated in Fig. 8.

## 5 Sweeteners

Increased sugar intake is widely recognized as a contributing factor to various health and nutritional issues, including obesity. The sweet taste of chemical compounds, whether naturally occurring or artificially synthesized, plays a crucial role in their use as sweetening agents. These compounds are commonly added to foods and beverages to enhance flavor, but their excessive consumption is linked to negative health outcomes (Mora & Dando, 2021). For thousands of years, the human diet has included a variety of natural sugars derived from berries, fruits and honey. Similarly, advancements in technology, such as the use of flexible, wireless in situ optical sensing systems, have allowed for precise monitoring of fruit ripening (M. Wang et al., 2023). High-intensity sweeteners, which are 50–100 times sweeter than sucrose, form a significant category of low-calorie sugar substitutes that have been investigated as potential alternatives to traditional sugars (Wierzbicka, 2021). However, sucrose took over as the primary sweetener utilized by consumers and the food industry around the beginning of the 20th century. Table sugar is currently produced in over 120 nations, with an annual global production of over 165 million tons 80 % from sugar cane and the remaining portion from sugar beets. The most crucial components in the food industry are sweeteners. Sweeteners are frequently used as sugar replacements in Pakistan's baking industry. They provide a low-calorie alternative to traditional sugar, helping meet the growing demand for healthier baked goods while maintaining sweetness and flavor (P. Singh et al., 2020). Sweeteners is being used by the confectionery and baking industry both alone and in conjunction with sucrose to reduce the use of sugars that high in calories. As a result, sweetening agents whether they are naturally occurring or are added are crucial components of the human diet. Their origin natural or synthetic, texture (syrups or powders), technological purpose (fillers or sweeteners) and nutritional value (caloric or non-caloric) can all be used to categorize them (Saraiva et al., 2020).

### 5.1 Natural sweeteners

**Fig. 9:**
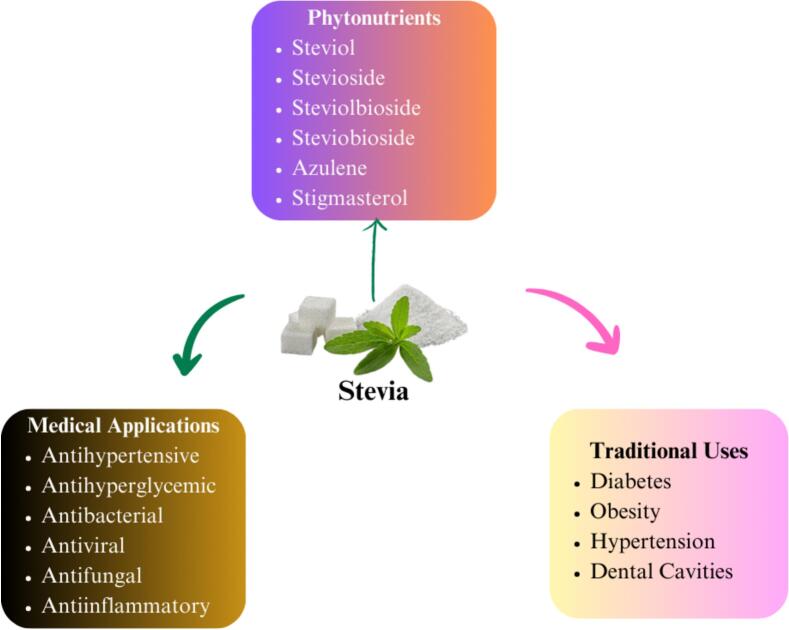
Stevia as a natural sweetener.

Researchers are exploring novel natural and synthetic sweeteners as alternatives to sucrose to meet rising demand (Saraiva et al., 2020). Natural sweeteners fructose, glucose and sucrose can be nutritionally helpful when derived from their sources as shown in Fig. 9. Valle et al. (2020) proposed and studied new options to meet demand such as erythritol, honey, maltose, maltodextrin, stevia, xylitol, molasses, maple syrup, agave nectar, coconut sugar and date sugar. The findings indicated that in diet-induced obese rats, natural sweeteners particularly molasses, agave syrup and maple syrup reduce the formation of insulin resistance and hepatic inflammation more than sucrose. This suggests that consuming those natural sweeteners is a less harmful choice than sucrose when it comes to obesity. The primary sweetener in the human diet used to be was honey. But in the 18th century, the method of obtaining sucrose from sugar cane and sugar beet increased rapidly and became evidently dominant. Nowadays, the most popular sweetener is still sucrose, sometimes referred to as standard table sugar, which is available in a variety of refined forms. A range of sweeteners are used in the baking industry to add sweetness, flavor and improve the texture and moisture content of baked goods. The following are a few typical sweetener varieties used in baking (Castro-Muñoz et al., 2022).

The granulated sugar (sucrose) is derived from sugar cane or sugar beets, this is the most used sweetener. It comes in granular form in fine, superfine and coarse varieties (Sahin et al., 2019). Because of its sweetness and useful qualities, sucrose is used in many culinary preparations. It helps preserve food and is essential to the structure of many foods such as ice cream, candies, biscuits and cookies (Van der Sman & Renzetti, 2019). The brown sugar has a rich flavor and a moist texture since it contains molasses. It is accessible in light and dark types; the molasses content of dark brown sugar is higher. Often used to provide moisture and depth of flavor to cookies, brownies and quick breads. Because of the molasses, brown sugar has a little higher mineral content than white sugar. These minerals include trace levels of potassium, calcium, iron and magnesium. These levels meanwhile are insufficient to have a meaningful impact on health (P. Singh et al., 2020). The honey is a natural sweetener made from floral nectar by bees. It tastes good and is abundant with minerals and vitamins. Additionally, honey includes trace levels of a number of substances that are thought to possess antioxidant properties such as catalase, vitamin C, pinobanksin, chrysin and pinocembrin. Because it retains moisture and has a flavor, it is used in baked goods and breads. It aids in browning as well (Talha et al., 2023). The maple syrup is a naturally occurring sweetener produced from sugar maple tree sap. It has several grades according to color and flavor intensity, and it has a distinct flavor. It is used for its unique flavor in breakfast pancakes, muffins and other baked items. Because maple syrup contains sugar, it encourages browning. This syrup infused baked items have a tendency to brown more quickly, so considerable observation of baking temperatures and durations may be required (Saraiva et al., 2022).

The molasses is a dark, potent residue of the refining process of sugar. Molasses is accessible in three shades: dark, light and blackstrap. Often used in ginger bread, spice cookies and other baked goods that need a strong flavor. Iron, calcium, magnesium and potassium are among the important minerals that can be found in good amounts in molasses. These nutrients are particularly abundant in blackstrap molasses. Because molasses has a modest glycemic index compared to refined sugar its effects on blood sugar levels are not as strong (Jamir et al., 2021). An agave nectar is a plant-based sweetener. It is sweeter than sugar and has a subtle flavor. Because of its special qualities, it can be used in baking and cooking for a range of purposes, from improving the texture and moisture of baked items to sweetening drinks. To get optimal results when utilizing agave nectar in recipes, bakers must adapt their measurements and methods. It should be used sparingly, taking into account dietary choices and personal health needs, much like any sweeteners (Ozuna & Franco-Robles, 2022). Another sugar substitute made naturally and without calories from stevia leaves. It's far sweeter than table sugar. Because stevia has a glycemic index of 1, it doesn't quickly raise blood sugar levels. It is used to temper its strong sweetness in baking and drinks, it is frequently mixed with other sweeteners. Because of its sweetening qualities and possible health advantages, stevia is frequently utilized in dietary supplements and health products (Šic Žlabur et al., 2013). A vast range of sweeteners are used in the baking business to create a variety of flavors, textures and health advantages in baked goods. Every sweetener, from contemporary low-calorie options to classic granulated sugar, has certain qualities that might improve baking results. Choosing the appropriate sweetener requires taking into account the particular needs of the recipe as well as the desired qualities of the finished product (Castro-Muñoz et al., 2022).

Stevia, agave syrup and honey all have distinct glycemic impacts. Stevia, a non-nutritive sweetener, has a glycemic index (GI) of 0, meaning it does not affect blood glucose levels, making it an ideal choice for those managing diabetes or blood sugar levels (Vaclavik et al., 2021). In contrast, agave syrup has a low GI of around 15–30 due to its high fructose content (70–90 %), which causes a slower rise in blood glucose. However, excessive intake of fructose can lead to metabolic issues like insulin resistance and fatty liver disease (P. Singh et al., 2020). Honey, with a moderate GI of 50–55, contains a mix of glucose and fructose, leading to a moderate spike in blood sugar. Despite its moderate glycemic impact, honey also offers small amounts of antioxidants, enzymes and vitamins, which can provide some health benefits (Rana et al., 2018). Thus, while stevia has no glycemic impact, agave and honey offer low to moderate glycemic responses, with agave being the most metabolically concerning when consumed in excess.

Farhat et al. (2019) investigated the impact of stevia on postprandial glucose levels, hunger, and food intake in a study involving 30 participants (20 females and 10 males; mean age 26.1 ± 10.56 years; BMI 23.44 ± 3.42 kg/m^2^). The study employed a three-arm crossover design where participants received preloads of water, sugar (60 g) and stevia (1 g) on three consecutive days, followed by an ad libitum pizza lunch to assess their responses. The findings revealed that Stevia reduces appetite feeling but does not increase consumption of food or postprandial glucose levels. It could be an effective technique for preventing and managing obesity and diabetes. Stevia can be used in candy and chocolates to accommodate diabetic demand while also preventing tooth decaying. Stevia's durability and sweetening strength make it a viable substitute to sugar in baking, but it must be handled carefully and frequently blended with other ingredients to obtain the proper texture and volume. Recent study Chen et al. (2024) showed that excessive fructose intake may play a significant role in the development of chronic kidney disease (CKD), with experimental evidence indicating that it can contribute to kidney injury. Catalpol, a bioactive iridoid glycoside derived from Rehmannia glutinosa, has demonstrated a range of pharmacological properties, including anti-inflammatory, antioxidant, and renoprotective effects. Notably, research suggests that Catalpol may help mitigate fructose-induced renal inflammation by inhibiting the TLR4/MyD88 signaling pathway and reducing uric acid reabsorption. In this context, alternative sweeteners such as stevia, monk fruit or erythritol can be effectively used in products made with composite flours to further reduce the glycemic impact, creating healthier baked goods.

## 6 Role of composite flours in baking industry

The role of composite flours in the baking industry extends beyond providing nutritional benefits and improving the glycemic index of products; they also influence the way sweeteners interact with the final product. Composite flour is made from several cereals such as barley, chickpeas, wheat, millet and other cereal grains. Consumers increasingly prefer ready-to-eat foods due to restricted time and modifications to their lifestyles (Banu et al., 2021; Cheng et al., 2023). Developing composite flours allows producers to sustain their crops. Increased urbanization and changing lifestyles have led to increased utilization of flour for bread and other bakery products. Blended flours in nations that are developing are both nutritious and cost-effective due to their use of low-cost ingredients (Sachdev & Misra, 2023). Composite flours replace wheat flour and reduce wheat imports, making them a cost-effective alternative. This kind of flour can be used in confectionary items however it lacks vital amino acids like threonine and lysine found in wheat and pulse flours (Olakanmi et al., 2022). Combining these flours improves both nutritional value and cost-efficiency. Adding flour to a composite food improves its emulsion stability, swelling ability and bulk density. Composite flour improves the nutritional content and taste of baked goods while also reducing the risk of chronic illnesses linked to contemporary lifestyles (Mughal, 2019).

**Fig. 10:**
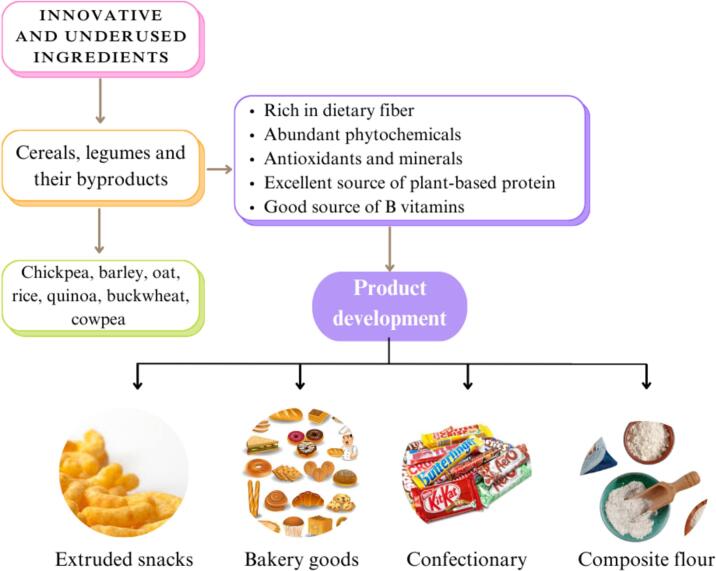
Exploring novel ingredients; cereals and legumes benefits and their applications in potential product development.

Composite flours are primarily used in bakery and pastry production to meet people nutritional needs, improve protein supply and minimize costs in nations that are developing by replacing wheat flour imports. Utilizing composite flours in bakery and pastry items improves nutritional content and amount of protein, as wheat is low in vital amino acids and regarded nutritionally inferior. Soybean, cassava and sorghum flours provide significantly more protein than wheat flour (Abioye et al., 2011; Banu et al., 2021). The nutritional content of bakery products varied depending on the value-added substances used. The widespread application of composite flours in bread, extrusion, confectionary and bakery products has led to more research on the impact of various flour ingredients on food product's physiochemical and functional properties as displayed in Fig. 10. Composite flour products provide substantial nutritional and health benefits, making them beneficial for individuals managing conditions such as diabetes, obesity, and constipation, as well as for those who are health-conscious. By combining different types of flours such as those made from whole grains, legumes or tubers, composite flours enhance the fiber, protein and micronutrient content of baked goods, contributing to improved digestion, better blood sugar control and weight management. Multigrain are commonly used in bread and breakfast cereals to improve the texture and taste, as well as increase acceptability and health advantages (Kapoor & Haripriya, 2020). They play a function in reducing cardiovascular disease, preventing diabetes, losing weight and improving digestion.

To optimize composite flour formulations made from cereals and legumes for improved texture, taste and glycemic control, several factors need to be considered. For texture, legume flours like chickpea and lentil are beneficial due to their high protein content, which enhances dough elasticity and improves chewiness when used in blends of 20–30 % This balance supports better product texture while maintaining a lower glycemic index, contributing to both nutritional benefits and consumer satisfaction (Olakanmi et al., 2022). Additionally, adding hydrocolloids like xanthan gum (0.5–1 %) and resistant starch (10–15 %) can help retain moisture, resulting in a softer, moister product and reducing crumbliness. Regarding taste, the earthy flavor of legume flours can be balanced by using a blend of 10–20 % legume flour with cereal flours like wheat or oats, which helps neutralize the flavor. Natural flavor enhancers such as citrus zest, vanilla or spices like cinnamon can further improve taste, while natural sweeteners like stevia or monk fruit offer sweetness without raising the glycemic index (Derseweh & Lauritsen, 2020). For glycemic control, legume flours are rich in fiber, which slows the digestion of carbohydrates and lowers the glycemic index (GI) of the final product. A blend of 30–40 % legume flour with whole grains, such as oats or barley, can reduce the GI by 20–30 %, while the addition of resistant starch (5–10 %) and prebiotics like inulin further enhances blood sugar regulation. By carefully balancing these ingredients, composite flours can be formulated to produce bakery products that offer improved texture, better taste and enhanced glycemic control, providing both culinary appeal and health benefits (Banu et al., 2021).

Noor Aziah et al. (2013) demonstrated that incorporating chickpea and mung bean flour into wheat flour increases protein and resistant starch content without altering the flour's functional properties. The study revealed significant differences in ash, fiber, protein, and total carbohydrate across the different cookie formulations. Chickpea cookies had the highest protein and resistant starch content compared to the other varieties. Mung bean cookies, on the other hand, exhibited a greater diameter, height, weight, and spread ratio. In terms of texture, chickpea cookies scored significantly higher in hardness, crispiness, flexibility, gumminess, and chewiness. While there were noticeable differences in flavor, crispiness, and aftertaste between the chickpea and mung bean cookies, both types were similarly rated for overall acceptability. However, chickpea cookies stood out for their superior taste, crispness, and overall acceptance. According to Shazia Saeed et al. (2013) using a 90:10 ratio of plain wheat flour and sweet potato flour yielded good results with no negative effects on the sensory or physical properties of cookies. It has been found that the spread of cookies is closely connected with their ability to absorb water. Sweet potato flour has a larger water absorption capacity 2.38 mL/g than wheat flour 1 mL/g, hence it is expected to partition free water hydrophilic sites more quickly than wheat flour. Sweet potato flour was also found to improve the flavor and texture of cookies, as well as dramatically increase the product's dietary fiber and mineral profile. The role of composite flours in the baking industry is closely linked to their therapeutic properties, offering significant health benefits when incorporated into bakery products.

## 7 Therapeutic properties of composite flour in bakery products

These low-glycemic index products' hypoglycemic, hypocholesterolemia, anti-diabetic and anti-obesity properties make them therapeutically viable snack options for people with non- communicable diseases (Dega & Barbhai, 2023). The approaches highlight the critical role of bioactive substances in modulating cellular processes, whether in the context of cancer therapy or in promoting functional food properties for enhanced health outcomes (Zhao et al., 2024). These bioactive compounds can influence key biological pathways, contributing to both disease prevention and improved overall wellness.

### 7.1 Hypolipidemic and anti-obesity attributes

Fu et al. (2021) found that feeding obese rats a high-fat diet and supplementing them with banana resistant starch at low 1.25, medium 2.50 and high 5.0 g/kg doses for six weeks could prevent the rise in glucose 4.16–3.78 mmol/L relative to the control group of obese rats. The obese rats had decreased triglyceride, low-density lipoprotein levels 0.44–0.47, 1.45–1.61 and 0.49–0.33 mmol/L, respectively and total cholesterol, compared to control obese rats 0.65, 1.79 and 0.48. They found that banana resistant starch modified the gut microbiota by improving the ratio of *Bacteroidetes*/*Firmicutes* microorganisms. Rats administered banana resistant starch had lower serum levels of leptin and insulin 1.82–1.37 ng/mL and 11.51–9.29 U/L, respectively compared to control rats' insulin: 15.15 U/L and leptin: 2.10 ng/mL. Banana resistant starch enhanced the ghrelin hormone concentration in obese rats 0.76 m U/L to 0.79–0.92 m U/L. Obese rats had low levels of adiponectin 23.60 mg/mL, while banana resistant starch fed rats had higher levels 24.71–34.44 mg/mL. Adiponectin is associated with anti-diabetic and cardio protective properties. Banana resistant starch showed anti-obesity effects by modulating lipid and glucose metabolism, lowering leptin and insulin levels and enhancing healthy gut bacteria.

Nichenametla et al. (2014) found that RS4-enriched flours have hypolipidemic characteristics. In both male and female metabolic syndrome patients, the RS_4_ enriched composite wheat decreased total lipids by 7.2 % and low-density lipoprotein by 5.5 % after 12 weeks of consumption of both the control (RS: 2 g/100 g) and RF_4_ supplemented flours (RS: 25 g/100 g). The findings indicated that adding RS_4_ to diets on a regular basis may improve dyslipidemia and reduce the risk of metabolic syndrome and cardiovascular disease. This encourages the use of novel ingredients and carbohydrates for their medicinal benefits.

### 7.2 Hypoglycemic properties

Diabetics should incorporate low glycemic load and glycemic index items into their diets due to their hypoglycemic effects as shown in Fig. S1. Proper nutrition management is crucial for diabetics to maintain normal blood sugar concentrations (Eleazu, 2016). The recent snacking trend involves consuming processed, high-calorie items such as bakery products and fried items which lack fiber and nutrients and have a high glycemic index (Gerontiti et al., 2024). Efforts are underway to lower the glycemic index of certain snacks such as bakery products, by including nutrient-dense foods rich in fiber and bioactive substances, in addition to standard nutrients. Overweight and obese women who consumed white bread fortified with oat fiber insoluble fiber experienced better insulin sensitivity after three days. Serum insulin levels in the experimental group were 29.7 mmol/L compared to 32.3 mmol/L in the control group (Weickert et al., 2006). Miranda-Ramos and Haros (2020) found that adding chia 10 % amaranth 20 % and quinoa 4 % to wheat flour lowered the glycemic index of the improved bread by 85 % in comparison to the control bread 95 %. The inclusion of amaranth, quinoa and chia in the blend leads to a lower glycemic index due to their significant amount of fiber and lower starch level. Adding pseudo cereals lowered the hydrolysis of starch at 90 min in improved bread 68.1 % versus to control 84.6 %, resulting in a hypoglycemic effect (Dega et al., 2023).

Research indicates that low glycemic index foods can be developed by incorporating fruits, cereals and vegetable byproducts. For example, mango peel, which is rich in dietary fiber and phenolic compounds, has been utilized to reduce the glycemic index of various food products. This addition not only enhances the nutritional profile but also aids in managing blood sugar levels. Ajila et al. (2008) found that adding mango peel 5–20 % to biscuits boosted total dietary fiber content to 20.7 % as compared to 6.5 % for the control group. The 20 % mango peel included biscuits had higher polyphenol content (4500 μg GAE/g) compared to the control (540 μg GAE/g). Silva et al. (2016) found that feeding diabetic rats a diet containing 75 % unripe banana pasta significantly reduced hyperglycemia, triglyceride levels, and cholesterol compared to the control group. Dietary fiber or resistant starch (RS) can help lower the glycemic index of foods and exert a hypoglycemic effect. Another study showed that adding 34 g of RS4 to a nutrition bar reduced both insulin and glucose responses for up to 120 min after consumption, compared to puffed wheat bars, demonstrating the potential benefits of resistant starch in managing blood sugar levels. Rakmai et al. (2021) found that replacing rice flour with 10 % resistant maltodextrin and 25 % sucralose significantly lowered the glycemic index of pancakes to 51.9 for Jasmine and 49.3 for Sangyod, compared to controls at 60.8 and 58.2, respectively. Additionally, this substitution reduced calories (305.59 Kcal), carbohydrates (39.16 g) and increased dietary fiber (0.93 g) and protein (6.39 g) compared to the control (protein: 5.75 g, carbohydrates: 46.59 g, fiber: 0 g). These findings suggest that incorporating dietary fiber and low-calorie sweeteners can create low-glycemic foods suitable for individuals with non-communicable diseases.

The mechanistic aspects of glycemic control involve complex biological processes that regulate blood glucose levels, ensuring they remain within an optimal range for normal cellular function and overall metabolic balance (Cherkas et al., 2020). The hypoglycemic effect of legume-based products is largely due to their high fiber content, which slows carbohydrate digestion. Additionally, bioactive compounds like isoflavones in chickpeas enhance insulin sensitivity by modulating metabolic pathways involved in glucose uptake (Zhang et al., 2021).

### 7.3 Anti-diabetic properties

Consuming composite flour has been demonstrated to significantly reduce blood sugar levels and prevent glucose spikes in individuals with diabetes. Incorporating dietary fibers like β-glucan and arabinoxylan in composite flour can minimize blood glucose level as illustrated in Fig. S2. Composite flour has been linked to increased insulin sensitivity and efficiency, which may assist blood sugar levels and prevent resistance to insulin. Consuming composite flour has been shown to significantly lower serum glycosylated proteins, glycosylated albumin, lipoprotein cholesterol and serum lipid levels, contributing to improved metabolic health and better management of blood sugar and lipid profiles. Bamson and Eze (2022) designed experimental and a simple random sample procedure to choose 90 healthy albino rats weighing between 197 and 255 g. The trial lasted twenty-one days. Research revealed that diabetic rats fed a firm porridge made from a millet composite flour blend experienced a positive effect in reducing hyperglycemia. Millet's low glycemic index makes it ideal for diabetic diets as it does not cause a significant increase in blood sugar levels. Alagbe and Malomo (2024) recent study conducted on the flours and cookies that were tested for their composition, amino acid profiles, bioactivities antioxidant and anti-diabetic and consumer acceptance using standard protocols. The cookie samples showed anti-diabetic characteristics in vitro, inhibiting α-amylase and α-glucosidase activity when compared to acarbose, a commonly used antidiabetic medication. The cookie sample made from 60 % wheat and 40 % soybean flour was well-received by consumers. It has potential as a bioactive antioxidant and anti-diabetic agent for managing diabetes. These cereal-based composite flours, packed with fiber and protein offers significant health benefits over refined flour. Despite their potential to create nutritious and functional bakery products, these flours remain underutilized compared to their wheat-based counterparts. To fully harness their advantages, more research is needed to optimize product formulation, value addition and increase consumer acceptance. These composite flours are cost effective, accessible and sustainable for improving nutrition and food security.

## 8 Sustainability and accessibility of composite flours in different regions

A global perspective on legume and cereal-based bakery products must account for how regional dietary habits influence their consumption and impact on glycemic response and chronic disease management(S. Kumar et al., 2022). In many developing nations, legumes and cereals form the foundation of daily meals. For example, in South Asia, legumes like lentils and chickpeas are dietary staples, while in sub-Saharan Africa, cereals such as maize and sorghum are prevalent. These foods are often consumed in whole or minimally processed forms, resulting in lower glycemic responses and better management of conditions like type II diabetes. However, in industrialized countries, refining processes tend to increase the glycemic index of these products, reducing their health benefits (Almarshad et al., 2022).

Sustainable and accessible composite flours made from blends of cereals and legumes offer great potential for enhancing food security and nutrition, especially in low- and middle-income regions. By using locally available grains and legumes, these flours can be produced with fewer resources compared to refined wheat flour, lowering the environmental impact of food production (Noort & Renzetti, 2023). Additionally, composite flours are often more affordable, making them a viable option for economically disadvantaged communities. However, challenges such as limited awareness, insufficient processing infrastructure, and restricted market availability must be addressed to fully harness their benefits across diverse regions, as illustrated in Table 4.

## 9 Conclusion

This manuscript underscores the significant role of cereal and legume-based products in promoting metabolic health and alleviating the risk of chronic diseases, such as type II diabetes and cardiovascular diseases. The nutritional composition of these foods, including high fiber, resistant starch, plant-based proteins and bioactive compounds, contributes to improved glycemic control, enhanced insulin sensitivity and reduced inflammation, all of which are critical for managing obesity, metabolic syndrome and other related conditions. While the health benefits of these products are well-established, there remain challenges in optimizing their sensory qualities; taste, texture and appearance to meet consumer preferences without compromising their functional health properties. Addressing these sensory issues is essential for increasing their acceptance and making them a practical part of daily diets. Moving forward, it is crucial to conduct long-term clinical studies to evaluate the sustained impact of these products on chronic disease prevention and management, particularly in terms of glycemic control. Additionally, personalized nutrition approaches should be explored to better tailor these interventions to individual dietary needs and responses.

Conclusively, cereal and legume-based products offer a promising dietary strategy for reducing the global burden of chronic diseases. Continued research and innovation are essential for optimizing these foods, ensuring they remain both nutritionally beneficial and widely accepted by consumers, ultimately supporting healthier lifestyles and improved public health outcomes.

## CRediT authorship contribution statement

**Hiba Naveed:** Writing – review & editing, Writing – original draft, Supervision, Project administration, Conceptualization. **Waleed Sultan:** Writing – review & editing, Software, Project administration, Formal analysis, Data curation, Conceptualization. **Kanza Aziz Awan:** Writing – original draft, Methodology, Formal analysis, Data curation, Conceptualization. **Aysha Imtiaz:** Writing – review & editing, Writing – original draft, Methodology, Investigation, Formal analysis, Data curation, Conceptualization. **Sanabil Yaqoob:** Writing – review & editing, Writing – original draft, Supervision, Resources, Formal analysis, Data curation. **Fahad Al-Asmari:** Writing – review & editing, Resources, Investigation, Data curation, Conceptualization. **Ahmad Faraz:** Writing – review & editing, Writing – original draft, Resources, Investigation, Formal analysis, Data curation, Conceptualization. **Jian-Ya Qian:** Writing – review & editing, Writing – original draft, Supervision, Project administration, Data curation, Conceptualization. **Aanchal Sharma:** Writing – review & editing, Validation, Software, Formal analysis, Conceptualization. **Robert Mugabi:** Writing – review & editing, Visualization, Funding acquisition, Formal analysis, Data curation, Conceptualization. **Saqer S. Alotaibi:** Data curation, Formal analysis, Funding acquisition, Software, Validation, Writing – review & editing. **Gulzar Ahmad Nayik:** Writing – review & editing, Writing – original draft, Supervision, Project administration, Investigation, Formal analysis, Data curation.

## Declaration of competing interest

The authors declare that they have no known competing financial interests or personal relationships that could have appeared to influence the work reported in this paper.

## Data Availability

All the authors declare that if more data is required, then the data will be provided on a request basis.

## Appendix A Supplementary data

Supplementary material Image 1
