# Supplementary material for: Glycemic impact of cereal and legume-based bakery products: Implications for chronic disease management
Source: Food Chem X. 2024 Nov 1;24:101959. doi: 10.1016/j.fochx.2024.101959 (PMC11577150; doi:10.1016/j.fochx.2024.101959)
Supplement: Supplementary file 1 — Supplementary material [file mmc1.docx]

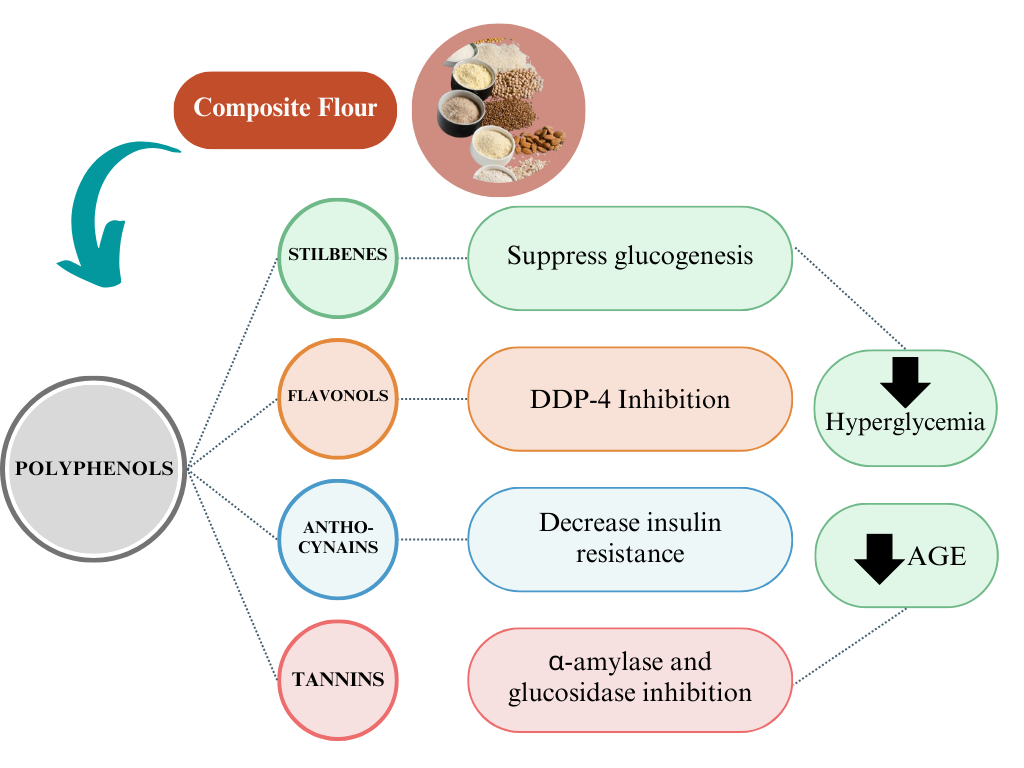


**Figure S1.** Composite flour polyphenols and their hypoglycemic pathways


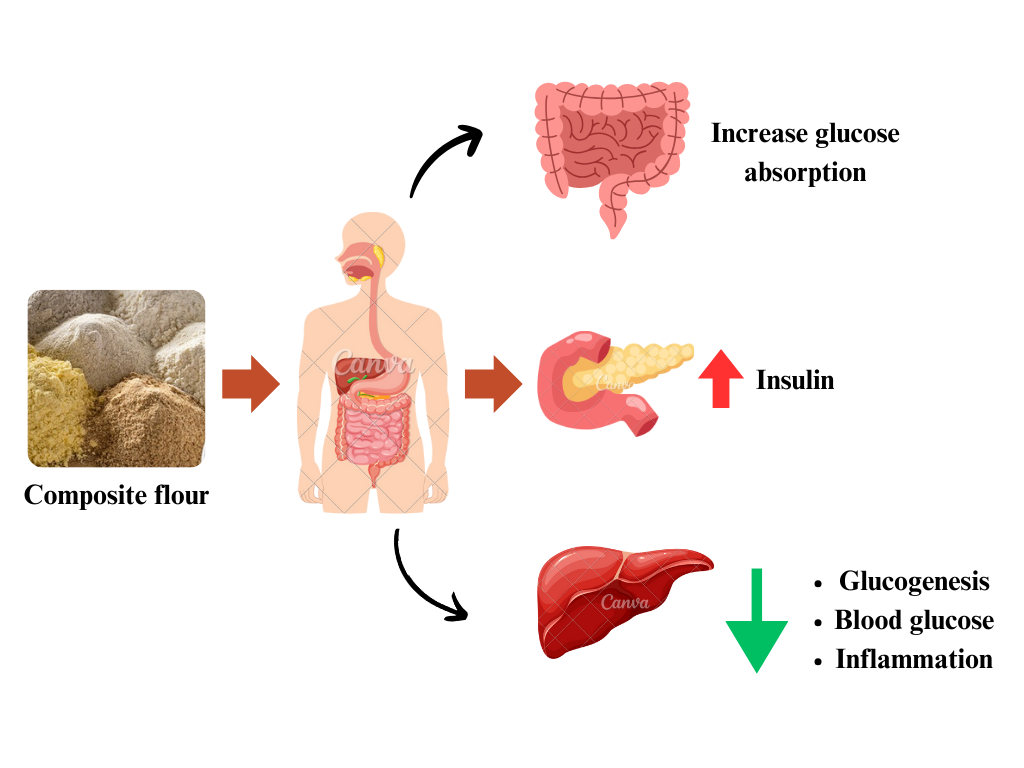


**Figure S2.** Antidiabetic effects of composite flour
